# Calcium hydroxide coating for orthopedic implants: evaluation of osseointegrative and antibacterial properties *in vivo*

**DOI:** 10.3389/fbioe.2025.1675881

**Published:** 2025-11-11

**Authors:** Sebastian Philipp von Hertzberg-Boelch, Andrea Ewald, Markus Meininger, Julia Ludwig, Fabian Vogt, Jürgen Groll, Maximilian Rudert

**Affiliations:** 1 Department of Orthopaedic Surgery, University of Wuerzburg, Wuerzburg, Germany; 2 Department for Functional Materials in Medicine and Dentistry, University of Wuerzburg, Wuerzburg, Germany

**Keywords:** infection model, antibacterial implant coating, bone–implant contact, implant osseointegration, periprosthetic infection

## Abstract

**Purpose:**

We evaluated the osseointegrative and antibacterial properties of calcium hydroxide-coated titanium implants in this study and compared them to uncoated implants in a rabbit model.

**Methods:**

Coated and uncoated implants were implanted into both femora of 19 New Zealand white rabbits. After retrieval, the osseointegrative properties were compared via quantification of bone–implant contact and proportion of unmineralized bone around the implant; further, the antibacterial properties were assessed using a *Staphylococcus aureus* infection model. The bacterial burden on and around the implants as well as the immunoreactions of the hosts were quantified using the neutrophil percentage in the tissue and effusion from the affected knees.

**Results:**

The bone–implant contact was significantly higher (*p* < 0.000) around the coated implant, whereas the proportion of osteoids was significantly higher around the uncoated implant (*p* = 0.001). The antibacterial effects of the coated implants were not significant. However, bacterial presence on the implant was observed in only 20% of the coated but 75% of the uncoated implants. The overall infection score indicated lower infection activities at joints with coated implants.

**Conclusion:**

Calcium hydroxide is a promising coating for titanium implants. Our animal study demonstrates the improved osseointegrative properties and evidences the topical antibacterial effects of coated implants.

## Introduction

Modern joint replacements lack implant surfaces that provide osseointegrative and antibacterial qualities; hence, new strategies are required for inhibition of biofilm formation (Simões and Simões, 2023). The strategies developed so far to prevent bacterial implant colonization are based on passive or active surface finishing methods and coatings (Davidson et al., 2019), while the strategies used to enhance osseointegration comprise modification of the implant surface or substrate as well as substrate surface treatment (Morrey et al., 2015). Finding a surface modification that combines both properties could be a game changer for preventing periprosthetic joint infection (PPI) in orthopaedic. Calcium hydroxide (Ca(OH)_2_) has been used in endodontics for more than 100 years because of its antibacterial properties and osteogenic potential (Mitchell and Shankwalker, 1958). Ca(OH)_2_ acts as a precursor for hydroxyapatite (HA) by supplying Ca^2+^ ions and hydroxyl groups (Kawashita et al., 2003). For example, HA is precipitated when phosphoric acid is titrated into a solution of Ca(OH)_2_ (Shah et al., 2004). HA has been used as a coating for orthopaedic implants (Gil et al., 2021; Soballe, 1993; Stephenson et al., 1991); here, the apatite layer functions as a provisional matrix for osteoblast adhesion and is subsequently resorbed by osteoclasts before being replaced with newly formed bone (Zhang et al., 2014).

Ca(OH)_2_ is also known to have antibacterial properties because conversion to HA releases hydroxyl (OH^–^) ions, which results in a pH change to alkaline condition (Siqueira, 2001). Both OH^–^ and the alkaline environment exert damage on bacterial DNA and the cytoplasmatic membrane, along with possible induction of protein denaturation (Mohammadi et al., 2012). Both hydroxyl and calcium ions are known to contribute to antibacterial effects against *Staphylococcus aureus* as elevated calcium levels can disrupt the bacterial membrane, likely owing to its specific lipid composition (Xie and Yang, 2016). Estrela et al. (2001) demonstrated the *in vitro* antibacterial effects of various Ca(OH)_2_ pastes against different microorganisms, including Enterococci and Staphylococci that are some of the predominant pathogens causing PPIs (Boelch et al., 2018).

In 2009, our study group published a technique for the electrochemical deposition of Ca(OH)_2_ onto titanium disks; these coated disks were tested *in vitro* and were proven to exert significant antibacterial effects (Moseke et al., 2009). The present study is a pilot *in vivo* investigation of the osseointegrative and antibacterial effects of Ca(OH)_2_ coatings on titanium implants. We also show the potential of Ca(OH)_2_-coated implants in an animal model by implanting both coated and uncoated titanium samples into rabbit femoral shafts. In an initial experiment, we assessed the osseointegrative properties of the coating by comparing the bony ingrowths of coated and uncoated implants. Then, in a second experiment, we investigated the antibacterial properties of the coating. Finally, the infection model was validated in a pioneering study to determine the cutoff values for infection markers. Thus, we demonstrated the feasibility of coated implants attenuating *S. aureus* infection compared to uncoated implants.

## Materials and methods

### Implant preparation

The implants used in this study were prepared according to the procedures described by Moseke et al. (2009). Briefly, dumbbell-shaped implants of dimensions 2 mm × 6 mm were prepared from medical-grade titanium; these implants were electrochemically coated with Ca(OH)_2_ and sterilized by autoclaving. Uncoated implants with identical features were used as the controls.

### Animal selection

The experiments reported herein were conducted after approval by the government of Lower Franconia as the responsible authority (reference number: 55.2-2531.01-19/11 for testing bone ingrowth (6 animals) and 55.2-2531.01-75/14 for the infection model (13 animals)). The sample size needed to evaluate osseointegration was determined by referencing previous studies on bone formation around coated and uncoated implants in rabbits (Wang et al., 2015). The infection model was designed to assess the ability of the coating to reduce the mean infection rate. In the control group, we anticipated an infection rate of approximately 75% (Arens et al., 1996), consistent with earlier reports of 47% at 5 × 10^4^ colony forming units (CFU) (Gosheger et al., 2004) or 40% at 10^3^ CFU and 70% at 10^5^ CFU (Cordero et al., 1994). Thus, three inoculation doses (10^3^, 10^4^, and 10^5^ CFU) were used to determine the mean infection rates in both the experimental and control groups. Each group was composed of five animals for every inoculation dose. In the pilot study, eight animals were included in the group. Hence, a total of 19 Harlan New Zealand white rabbits were used in the experiments. The average weight of each animal was 2,900 g, and the animals were housed in open cages for 2 weeks prior to the start of study with free access to water and food. After the surgical implantation procedure, the rabbits were supplied with analgesics and monitored daily for changes in their weight, body temperature, and wound situation until euthanasia.

### Bacterial inoculum preparation

The *S. aureus* (strain RN4220) used in this study was cultivated in LB-medium (10 g of pepton/trypton, 2 g of yeast extract, and 5 g of NaCl in 1,000 mL of water) under aerobic conditions overnight at 37 °C. The number of bacterial cells was adjusted to 1,000 in 10 µL by dilution in phosphate-buffered saline (PBS). This number was verified by determination of the CFU.

### Surgical procedure

**FIGURE 1:**
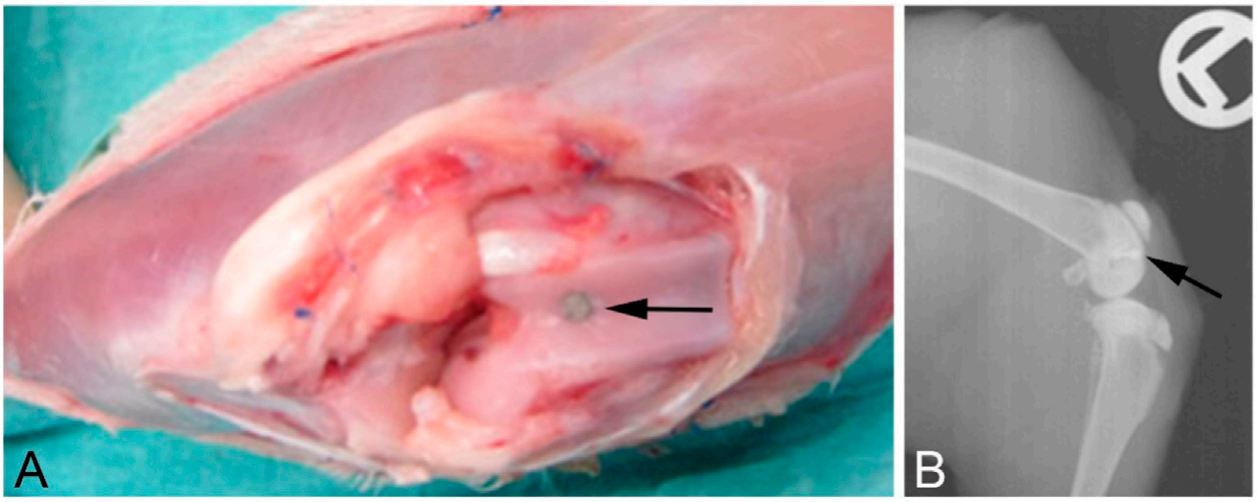
**(A)** Surgical situs directly after introduction of the implant. **(B)** X-ray after euthanasia showing the implant localization in the bone. The implants are indicated by the black arrows.

Anesthesia was induced via intramuscular injection of medetomidine, midazolam, and fentanyl adjusted to the bodyweight (0.2 mg/kg, 0.4 mg/kg, and 0.3 mg/kg, respectively) and was maintained using isoflurane 0.3% in oxygen along with monitoring by a veterinarian. After surgery, the anesthesia was reversed using atipamezole, flumazenil, and naloxone adjusted to the bodyweight, and analgesia was maintained via oral administration of meloxicam. Briefly, both hind legs of the animals were shaved and prepared with polyhexanide before applying sterile dressings. To analyze the osseointegrative properties of the implant coating, probes were placed into each femoral shaft. Therefore, the skin was incised at the extensor side of the lateral thigh up to the thigh extensor muscles (M. quadriceps femoris), followed by preparation within the muscle septa on the femur. After incision and preparation of the periosteum, three cylindrical holes of 2 mm diameter × 6 mm depth were drilled on each side in the area of the femoral diaphysis. For this purpose, we used a commercially available maxillofacial trephine drill at low speed with continuous cooling (using physiological saline solution). After creating and rinsing the drill holes, the implant bodies were inserted conclusively. Then, a two-layer subcutaneous and cutaneous wound closure was performed using vicryl 3-0 single button sutures. Finally, the wound was sealed with a spray dressing. To evaluate the antibacterial effects of the Ca(OH)_2_ coating, probes were placed in the femora at the knees; here, the skin over the knee was incised, followed by a medial parapatellar arthrotomy. A hole was drilled with a trephine burr of 2 mm diameter to a depth of 6 mm. Then, the implant was introduced flush with the subchondral bone of the trochlea (Figure 1).

**FIGURE 2:**
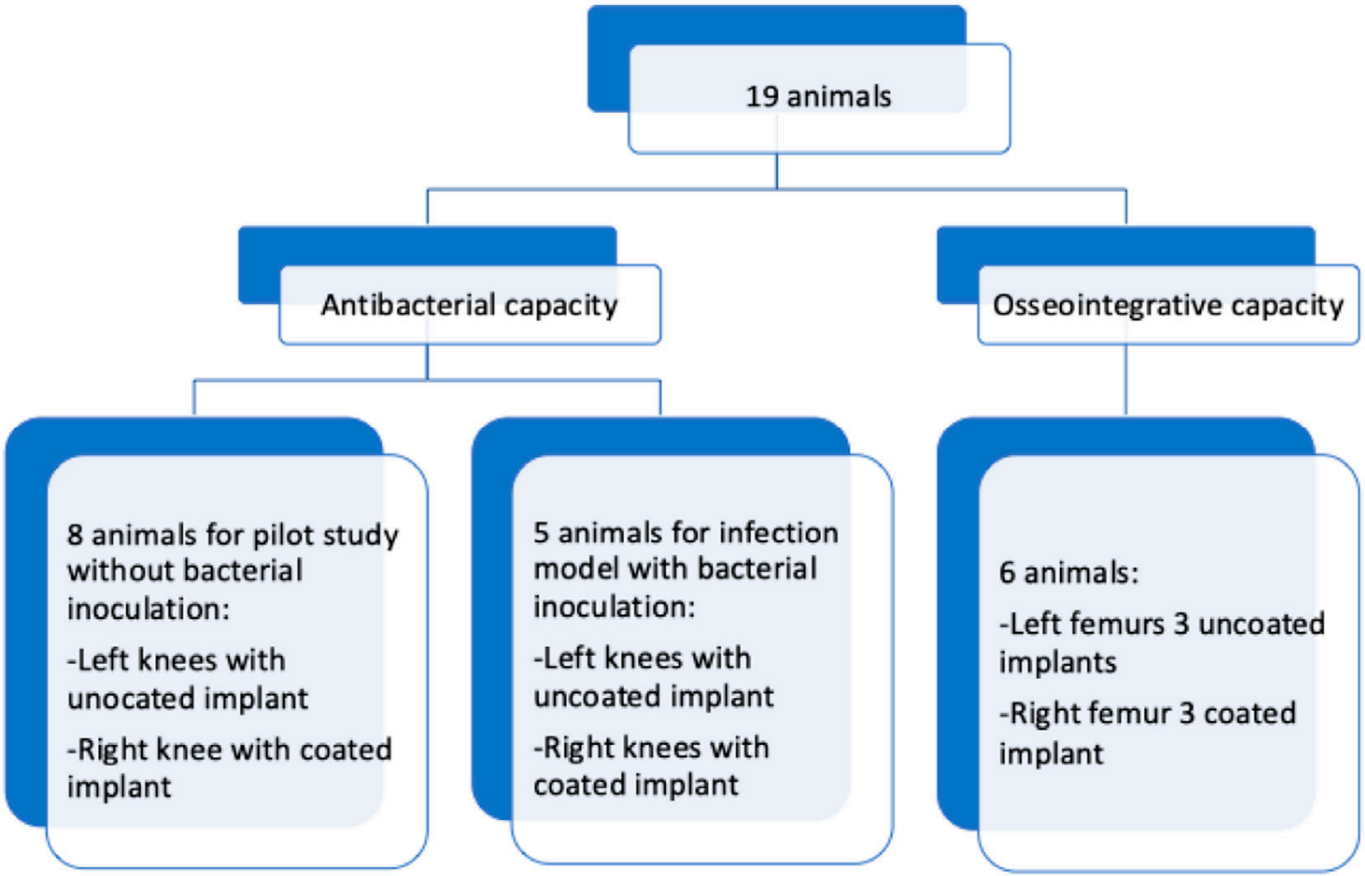
Overview of animal allocation for the experiments.

After placing the implant, the two-layer subcutaneous and cutaneous wound closure was performed using vicryl 3-0 single button sutures. The skin was then stitched with 7-0 non-resorbable sutures. In the eight animals used in the pilot study, NaCl was first injected into the drill holes, followed by insertion of an uncoated control implant in one knee and insertion of a coated implant in the other knee. For the infection model, identical bacterial suspensions (1,000 CFU each) were injected into both knees at the drill holes, followed by insertion of a coated implant on one side and insertion of an uncoated control implant on the other side (five animals for each infection model). Figure 2 depicts the allocation of animals for the experiments.

### Analysis of osseointegrative properties

Twelve weeks following surgery, the animals in this group were euthanized with an overdose of phenobarbital after anesthesia using ketamine and xylazine adjusted to the bodyweight; all procedures were performed by a veterinarian. The femora were then removed, and the area of implantation was placed in 10% formalin for fixation. The samples were cut into blocks, dehydrated using glycol methacrylate, and stabilized with Technovit (Heraeus Kulzer). Thin specimen sections of 11–26 µm were then produced and stained using the trichrome Masson-Goldner technique described by Donath (1988). The bone–implant contact (BIC) and proportion of osteoid were quantified using ImageJ software (National Institutes of Health, Bethesda, MD, United States). The scale bar in the overview image was used to calibrate ImageJ, which yielded a ratio where 310 pixels corresponded to 100 μm at 20× magnification. Freehand lines drawn with the magnification tool were used to determine the region of interest, which included the implant perimeter and BIC. Border areas with ambiguous tissue quality were examined under a EUROMEX CKL microscope at up to ×640 magnification (×40 objective and ×16 ocular). The verified tissue classification was then converted to a panoramic image in ImageJ and measured.

### Analysis of antibacterial properties

The animals in this group were euthanized after 7 days by administration of an overdose of phenobarbital (200 mg/kg intravenously) after anesthesia using ketamine and xylazine adjusted to the bodyweight (30 mg/kg and 3 mg/kg intramuscularly); these procedures were performed by a veterinarian. The presence of purulence, presence of a sinus tract, and macroscopic extension of infection graded as mild, along with moderate and severe inflammation at the knees were documented in the animals. After sterile draping of both hind legs, the knee was reopened using the earlier approach; then an effusion sample was obtained for leucocyte counting and bacterial culturing. Next, tissue samples were collected for CFU counting (bacterial evaluation) and fixed in 10% formalin before embedding in paraffin for leucocyte counting and histological analysis. Additionally, the retrieved implants were transferred to vials containing 500 µL of sterile 0.9% NaCl and subjected to sonication in an ultrasonic bath (Bandelin Sonorex RK 512 H, Bandelin Electronic, Berlin, Germany) for 30 s at 35 kHz and room temperature; this treatment procedure was repeated 10 times with paused of 15 s between the cycles. The samples were next vortexed four times using a Vortex Genie 2 (Scientific Industries, New York, United States) at level 9 for 15 s each, along with 45-s pauses between consecutive vortexing steps. Finally, 100-µL aliquots of serial 10-fold dilutions of the resulting solution were plated onto LB agar plates. An overview of the sample collection and evaluation is depicted in Table 1.

**TABLE 1:** Collected samples and their evaluation methods.

Type of sample	Type of evaluation
Effusion	Leukocyte counting and differentiation (smear)
Intraarticular tissue	Leukocyte counting and differentiation (section)
Intraarticular tissue	CFU counting after homogenization (culturing)
Implant	CFU counting after sonication (culturing)

### Paraffin section preparation

After fixation in 10% formalin and washing thrice with PBS for 10 min each, the samples were automatically embedded in paraffin wax (Microm STP 120, Thermo Scientific) according to manufacturer instructions. The sections were then prepared using a rotary microtome (MicroTec Cut 4060) and stained as described below.

### Infection activity scoring

The infection activity scoring developed in this work is as follows. One point each was assigned for the presence of a sinus tract, purulence, bacterial growth in the tissue sample, bacterial growth on the implant, and mild inflammation. Two points each were assigned for neutrophil percentages exceeding the cutoff value (determined in the pilot study) for the tissue/effusion smears and for moderate inflammation. Three points were assigned to severe macroscopic inflammation. Thus, the infection activity score for each animal ranges from a minimum of 0 to a maximum of 9.

### Sample processing for CFU counting, leucocyte counting, and differentiation

After opening the knees, the synovia were aspirated and 10 µL of the fluid was diluted in 40 µL of sterile 0.9% NaCl; this suspension was pipetted onto agar plates (containing 10 g of pepton/trypton, 2 g of yeast extract, 15 g of agar, and 5 g of NaCl in 1,000 mL of water) for CFU counting after incubation for 24 h at 37 °C. The tissue samples were homogenized in 150 µL of 0.9% NaCl, and the solution was diluted stepwise in 0.9% NaCl before being plated on agar plates. The implants were transferred to vials containing 500 µL of sterile 0.9% NaCl and subjected to several rounds of treatment in an ultrasonic bath and vortex. The as-obtained solutions were then diluted stepwise in sterile 0.9% NaCl and plated on agar plates for CFU counting. To analyze the cell composition, 20 µL of the synovial fluid was pipetted onto a glass slide; the liquid was then scratched over the slide using the edge of another slide. For identification and quantification of the different cell types, the samples were stained according to the Pappenheim method. Lastly, the paraffin sections were deparaffinized in xylene, followed by rehydration in a series of descending ethanol baths and H&E staining.

### Statistical evaluation

A mixed-effects model was used to compare the BIC and osteoid proportions, where a generalized linear model (subject variable: experimental animal; within-subject variables: investigation site and investigation group) was applied to each target variable (BIC and osteoid proportions) using the Wald chi-squared test. For the infection model, the samples were assessed for distribution using the Kolmogorov test. The t-test was used for normally distributed samples, and the Mann–Whitney U test was used for non-normally distributed samples. The level of significance was set at *p* < 0.05. The normally distributed data were displayed as mean values and range, while the non-normally distributed data were quantified as median values with interquartile ranges. All statistical evaluations were performed with SPSS 25 (IBM Corp., Armonk, NY, United States).

## Results

### Osseointegrative properties

The coated implants demonstrated significantly more BIC with a lower proportion of osteoid, as depicted in Table 2.

**TABLE 2:** Bone–implant contact (BIC) and osteoid proportion sorted by uncoated and coated implants.

	Uncoated implant	Coated implant	*p*-value
BIC in mean (min–max)%	85.0 (75.5–95.7)	88.8 (83.8–95.1)	<0.001
Osteoid proportion %	56.95 (21.45–76.03)	32.3 (21.45–42.64)	<0.001

**FIGURE 3:**
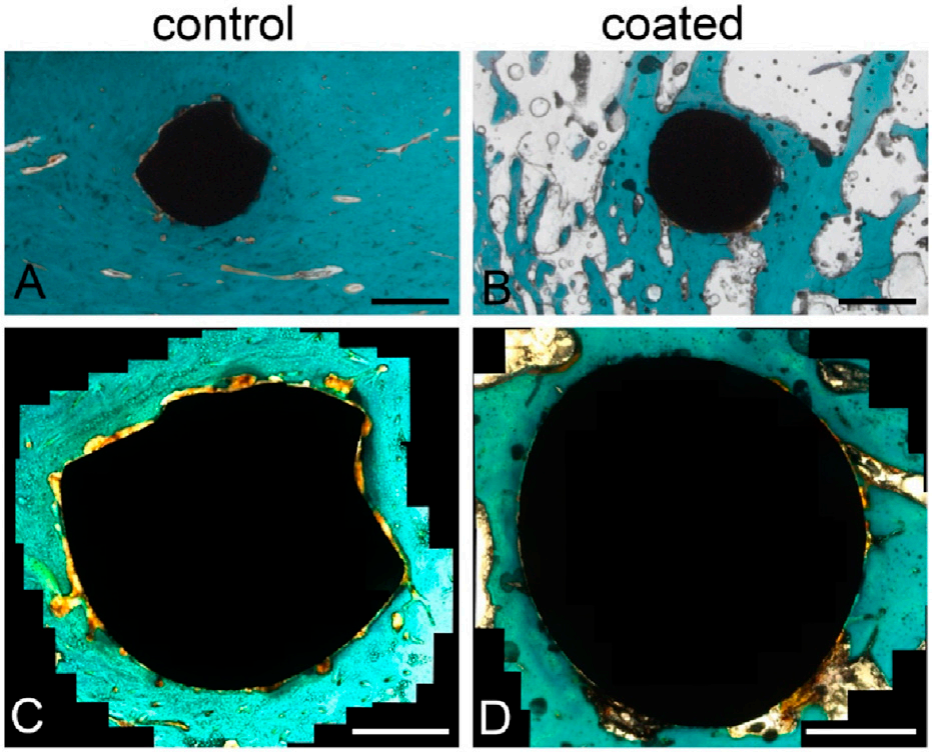
Trichrome Masson–Goldner staining of the histological sections to evaluate bone–implant contact (BIC). Overview of a slice with **(A)** an uncoated implant and **(B)** a coated implant. Magnified images of the **(C)** uncoated and **(D)** coated implants showing areas of not-yet-calcified bone (osteoid, orange) and close contact between the bone tissue (dark green, orange) and implant. Scale bars: 1 mm **(A,B)**, 500 µm **(C,D)**.

Representative examples for the BIC and presence of osteoid after staining are shown in Figure 3.

### Antibacterial properties

#### Pilot study

The mean neutrophil percentages were significantly higher in the effusion and tissue sections from knees with uncoated implants rather than the coated implants, as shown in Table 3.

**TABLE 3:** Results of the effusion and tissue smears from the pilot study.

	Knees with coated implants (neutrophils in %)	Knees with uncoated implants (neutrophils in %)	*p*-value
Effusions smears	6.3 (0.0–12.16)	15.2 (27.5–4.9)	0.037
Tissue smears	2.9 (5.5–0.4)	15.6 (34.8–3.3)	0.015

We could not culture bacteria from the effusion or tissue samples in the pilot study as all samples were sterile.

### Infection model

We did not observe significant differences in the neutrophil numbers for the effusion and tissue sections sorted by control and probe (experimental) groups, whose results are summarized in Table 4.

**TABLE 4:** Results of the effusion and tissue smears sorted by control and probe groups.

	Control (neutrophils in %)	Probe (neutrophils in %)	*p*-value
Effusions smears	82.6 (74.1–89.3)	83.5 (69.5–90.3)	0.800
Tissue smears	58.2 (15.7–83.6)	43.3 (5.8–56.8))	0.496

In the experimental group, three of the five animals had lower mean neutrophil percentages in the tissue samples and effusion from the knees. The plating and CFU counting analyses showed lower mean numbers of bacteria in the tissue samples from knees in the experimental group (Table 5). Sonication of the implants showed no significant differences in CFU numbers. Although bacterial growth was found on 75% of the samples in the control implants, only 20% of the experimental implants showed bacterial growth.

**TABLE 5:** Results of CFU counting from the tissues and implants sorted by control and probe groups.

	Control mean (range) (N)	Probe mean (range) (N)	*p*-value
Tissue mean (CFU/mg)	2.16 (0.02–70.01) (10)	2.80 (0.31–37.42) (10)	0.917
Implant	0 (0.00–22.5) (5)	0 (0.00–20.8) (5)	0.906

### Infection activity score: derivation of neutrophil cutoff value

**FIGURE 4:**
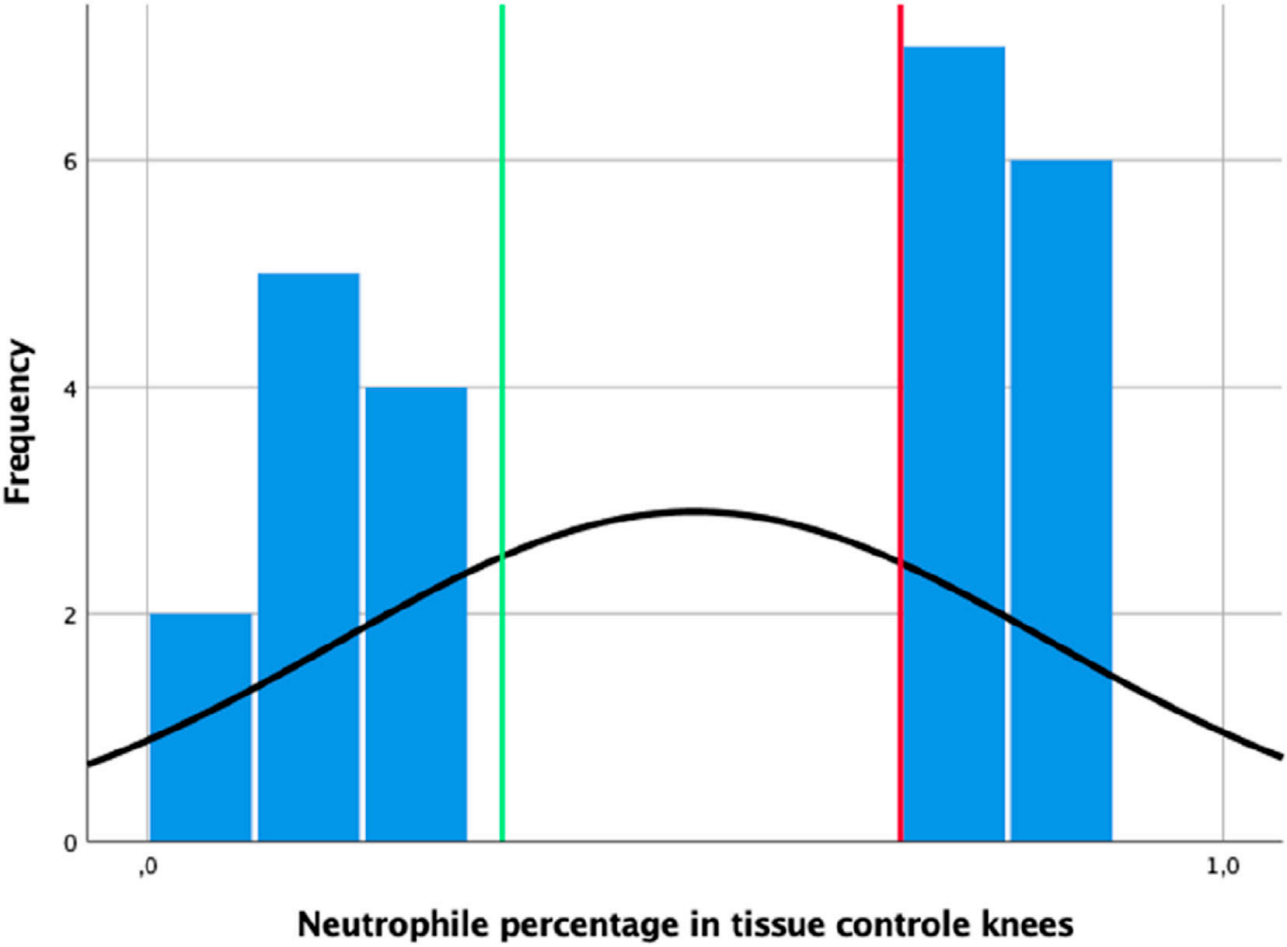
Distribution of neutrophil percentages observed in the tissue sections of the control knees; the vertical green line indicates the 0.35 mark, while the vertical red line indicates the derived cutoff value of 0.7.

The results of neutrophil percentages in the control animals, i.e., inoculation but no coating, can be stratified under two groups; here, the first group has a maximum of 35% neutrophils (left of the green line in Figure 4), while the second group has a minimum of 70% neutrophils (right side of the red line in Figure 4). In knees with coated implants from the pilot study, the highest percentage of neutrophils in a single tissue section was 5.5%. Thus, a cutoff value of 0.7 was derived for tissue neutrophil percentages indicating infection.

The infection activity scores shown in Table 6 indicate stronger infection in the control animals under all evaluation categories.

**TABLE 6:** Distribution of points for the infection activity scores of individual animals in the experimental and control groups.

Animal	Sinus tract		Purulence		Inflammation		Neutrophil percentage in tissue > 0.7		Bacterial growth on implant		Bacterial growth in tissue		Sum	
	C	P	C	P	C	P	C	P	C	P	C	P	C	P
1	1	0	1	0	3	1	2	0	1	0	1	0	9	1
2	0	1	0	0	2	3	2	0	1	0	1	1	6	5
3	1	0	1	1	3	2	0	0	0	0	1	1	6	4
4	0	0	1	1	2	1	2	0	0	1	1	1	6	4
5	0	0	1	1	2	2	2	0	1	0	1	1	7	4
Overall score	2	1	4	3	12	9	8	0	3	1	5	4	34	18

C: control; P: probe; Ratings: presence of sinus tract (1 point); presence of purulence (1 point); presence of inflammation (mild: 1 point, moderate: 2 points, severe: 3 points); neutrophil percentage > 0.7 (2 points); bacterial growth on implant (1 point); bacterial growth in tissue (1 point).

## Discussion

The aim of the present study was to investigate the osseointegrative and antibacterial properties of a new Ca(OH)_2_ coating for titanium implants. Accordingly, the experiments comprised evaluations of implant ingrowth, reduction of infection activity, and bacterial burden at the implantation site. The osseointegrative properties were found to be better for the coated than uncoated implants. Moreover, the antibacterial properties of the coating tended to reduce bacterial colonization on the implant but could not prevent septic arthritis. All knees showed purulence and high neutrophil percentages in the smears from the effusion samples. The bacterial inoculum was injected at the drill holes, and spilling of this inoculum into the knee joint was intended to mimic intraoperative contamination that is always observed during implant introduction. The bacterial inoculation dose used herein was extrapolated from previous animal infection models. One of the lowest inoculum doses used in literature was reported by Cordero et al. (1994), who noted an infection rate of 50% upon inoculating only 2,370 CFUs into the femora of New Zealand white rabbits. In contrast, one of the highest inoculation doses used was reported by Arens et al. (1996), who noted an infection rate of 50% around a plate fixed to a canine tibia upon inoculation with 2 × 10^5^ CFUs. Qiao et al. (2025) demonstrated a correlation between infection severity and inoculation dose for *Mycobacterium tuberculosis in vitro*. Thus, the minimum bacterial inoculation dose necessary to produce an infection is dependent on not only the antibacterial effect of the implant but also on the experimental setup.

In the current study, only the surface of the dumbbell-shaped part of the implant was in contact with the knee joint. The implant coating typically exerts its antibacterial properties by alkalization of the implant surroundings (Siqueira, 2001; Mohammadi et al., 2012). Thus, the antibacterial effects were not strong enough to prevent infection of the knee. However, the coating is not intended to prevent septic arthritis but only to protect the implant from bacterial colonization. Our results indicate this topical antibacterial effect of the Ca(OH)_2_ coating. Although bacterial growths were observed in all the tissue samples from the infection study, bacterial colonization of the implants were found in only 20% of the coated samples versus 75% of the uncoated samples. Of note, the evaluation of the antibacterial effects had to be discontinued as all animals developed an infection in the investigated joint. Nevertheless, the objective infection activity score indicated a weaker infectious process in the knees receiving coated implants. Although the study was underpowered owing to early termination and no statistically significant results, the findings suggest a potential antibacterial effect of Ca(OH)_2_ as coating for titanium implants. In the current study, *S. aureus* strain RN4220 was chosen because it is a laboratory strain that allows reproducibility; although this strain does not produce polysaccharide intercellular adhesin, which is a classical feature of biofilm-forming isolates, Lade et al. (2019) demonstrated its potential for biofilm formation under suitable conditions. Nevertheless, future studies should focus on clinical isolates, could include administration of appropriate antibiotics, and may consider implant contamination before insertion into the drill hole rather than introducing contaminants directly into the drill hole to improve translational applicability.

While CFU counting remains the standard for quantitative biofilm evaluation (Thieme et al., 2021), its variability may reflect the presence of viable but non-culturable bacteria (Fabritius et al., 2020). Culture-independent methods such as qPCR (Magalhaes et al., 2019) could offer greater sensitivity but require combination with approaches that can distinguish live from dead cells. Instead of comparing the mean CFU values, cutoff values of neutrophil percentages from periprosthetic tissues are often used to define the presence of PPIs in human arthroplasty (Krenn et al., 2014; Luppi et al., 2023); this approach was also transferred to the present animal study, where the cutoff value was derived from the pilot study. All tissue samples from around the coated implants showed neutrophil percentages below the cutoff value, compared to only two samples from the uncoated implants. However, the pilot study showed significantly lower neutrophil percentages in knees with coated implants. Thus, reduction of inflammatory cellular responses owing to the presence of the coating rather than reduction of infectious activity itself may be another explanation for the results obtained herein.

Antibacterial surface modifications have demonstrated both efficacy and biocompatibility, and several of these approaches are already in clinical use. Antibiotic-releasing surfaces show antibacterial activities in animal models without impairing osseointegration (Alt et al., 2011). One such example is the defensive antibacterial coating (DAC^®^), which is a fast-resorbable hydrogel that serves as a local drug-elution matrix. In a randomized prospective trial of 380 patients undergoing total hip or total knee replacements, DAC was shown to reduce early infections significantly without compromising osseointegration (Romanò et al., 2016). Among antibacterial implant coatings, silver is one of the most extensively studied and clinically established materials; its antibacterial activity has been confirmed in preclinical studies. For example, in a rabbit megaprosthesis infection model, silver-coated implants reduced *S. aureus* infections to 7% compared to 47% for uncoated titanium, while also lowering the inflammatory marker levels. Importantly, no histological organ damage was observed despite measurable silver concentrations in the blood and tissues (Gosheger et al., 2004). Silver-based coatings are also in clinical use currently. For instance, silver hydroxyapatite hip implants were reported to improve functional outcomes at 5-year follow-up without the adverse effects attributable to silver (Kawano et al., 2022). Nevertheless, there remain concerns regarding the potential toxicity of silver ions (Glehr et al., 2013). In contrast, Ca(OH)_2_ coatings represent a novel approach: unlike silver, they are intrinsically osseointegrative and combine antibacterial action with enhanced bone integration.

Moseke et al. (2009) described nearly complete conversion of Ca(OH)_2_ to HA after 3 days *in vitro*; this conversion is the most likely reason for the significantly higher proportion of mineralized bone around the coated sample. Today, HA-coated implants are routinely used clinically (Chambers et al., 2007); radiographic studies following their clinical application as total hip-joint replacements show improved osseointegration of these coated implants. Animal studies have also demonstrated improved osseointegration antecedent to successful clinical application. For example, Roy et al. (2019) reported improved bone ingrowth onto HA-coated titanium implants than uncoated implants in the femora of rats. Junker et al. (2010) investigated the bone bonding of HA-coated implants after 6 and 12 weeks in goats via torque measurements; irrespective of the implant material used, HA-coated implants showed better bone bonding than uncoated implants as HA accelerates the mineralization of organic bone matrix (Gil et al., 2021). Similar to the present study, implant osseointegration has been evaluated under non-load-bearing conditions. However, translation of the osseointegration outcomes to human application requires investigation under functional loading since *in vivo* loading has been shown to positively influence implant fixation, particularly in the case of HA-coated implants (Mouzin et al., 2001). Therefore, evaluating implants without assessing the load-bearing capacities may underestimate their true performances in clinical situations.

## Conclusion

This study demonstrates that Ca(OH)_2_ coating of an implant promotes better osseointegration. Furthermore, the results indicate that the antibacterial effects of this material observed from dental surgery outcomes may also be reaped when used as a coating for titanium implants in bone. Thus, in endoprosthetics, this coating has the potential to extend the longevity of a prosthesis as well as reduce the risk of postoperative implant infections.

## Data Availability

The raw data supporting the conclusions of this article will be made available by the authors without undue reservation.
